# COVID-19 Illness in a Patient With Anti-Glomerular Basement Membrane Disease: A Clinical Dilemma

**DOI:** 10.7759/cureus.14912

**Published:** 2021-05-08

**Authors:** Vineeta Venkateswaran, Apoorv Chaturvedi, Kapil D Soni, Richa Aggarwal, Anjan Trikha

**Affiliations:** 1 Anaesthesiology, Pain Medicine and Critical Care, All India Institute of Medical Sciences, New Delhi, IND; 2 Critical and Intensive Care, Jai Prakash Narayan Apex Trauma Centre, All India Institute of Medical Sciences, New Delhi, IND

**Keywords:** covid-19, anti-glomerular basement membrane disease, therapeutic anticoagulation

## Abstract

Anti-glomerular basement membrane (anti-GBM) disease is a rare autoimmune disease affecting the kidneys and lungs. COVID-19 infection in a patient with pre-existing anti-GBM disease presents a unique set of clinical challenges. The formulation of a judicious treatment plan balancing both disease processes is tricky, especially with regard to anticoagulation. We present the case of a young patient with anti-GBM disease who acquired COVID-19 infection and eventually succumbed to his illness.

## Introduction

Anti-glomerular basement membrane (anti-GBM) disease is a rare small vessel vasculitis that affects the capillary beds of the kidneys and lungs [1]. COVID-19 infection in a patient with pre-existing anti-GBM disease presents a unique set of clinical challenges. We present the case of a young patient with pre-existing anti-GBM disease who acquired COVID-19 infection.

## Case presentation

**Figure 1:**
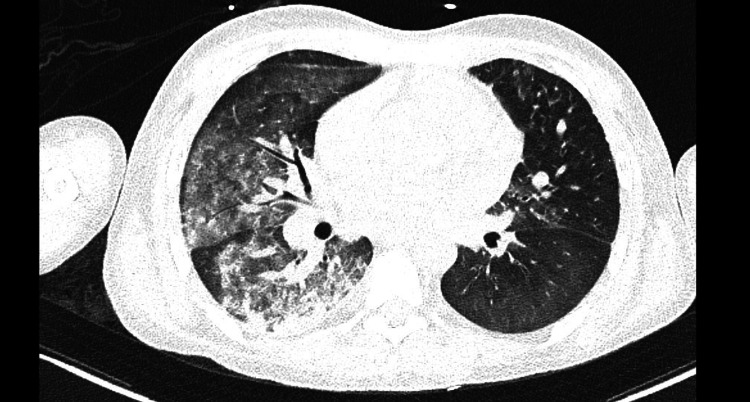
Computerised tomograph of the chest showing diffuse alveolar infiltrates suggestive of diffuse alveolar haemorrhage

A 19-year-old male patient presented to our institute with cough and dyspnoea. He had earlier been diagnosed as having anti-GBM disease, based on the presence of anti-GBM antibodies in the blood. He had end-stage renal failure, and was dialysis dependent and awaiting renal transplantation. In the preceding month, he had been hospitalised for two weeks for diffuse alveolar haemorrhage (DAH) and had been discharged from the hospital in stable condition. At presentation to our centre, the patient had significant dyspnoea with high oxygen requirement. Following a positive test for severe acute respiratory syndrome coronavirus 2 (SARS-CoV-2), he was immediately admitted in the intensive care unit (ICU). Chest radiograph at the time of admission indicated severe infiltration of the right lung and mild-to-moderate involvement of the left lung. High-resolution CT of the chest showed diffuse alveolar infiltrates predominantly in the right lung, suggestive of DAH (Figure 1).

Investigations were remarkable for a platelet count of 39,000 per microlitre and haemoglobin of 7.7 gm%, which necessitated transfusion of two units of packed red cells. He was administered oxygen via high-flow nasal cannula and broad-spectrum antibiotics, anticoagulant, pulse steroids (500 mg methylprednisolone twice a day for three days, followed by 20 mg twice a day), zinc, and vitamin C. As he was in advanced renal failure stage and had been anuric for the preceding two days, he underwent haemodialysis upon admission, and every alternate day thereafter. Haemodialysis was performed without the use of heparin considering the thrombocytopenia. Plasmapheresis was done on the fourth day of ICU stay, and another four cycles were planned over the next eight days. Over the next few days, he showed radiological and clinical improvement. Oxygen requirement reduced and the patient stabilized on oxygen via non-rebreathing mask. On day 6 of ICU stay, he suffered an episode of massive haemoptysis while undergoing haemodialysis. The trachea was immediately intubated, mechanical ventilation was initiated, and resuscitation with fluid and blood products was started. A repeat chest radiograph showed marked worsening, with nearly complete dense infiltration of the right lung. Arterial blood gas showed significant hypoxia even on mechanical ventilation with 100% oxygen. A few hours later, the patient succumbed to his illness.

## Discussion

The term ‘anti-GBM disease’ refers to the presence of anti-GBM antibodies in the blood along with renal and lung involvement [1]. Treatment includes plasma exchange therapy to remove the causative antibodies, in combination with steroids and cyclophosphamide to suppress the body’s immune response [1]. SARS-CoV-2, the causative agent of COVID-19 infection, elicits a severe inflammatory reaction in the lung epithelium and the pulmonary capillary endothelium. The influx of leucocytes, alveolar oedema, and hyaline membrane formation lead to disruption of the endothelial barrier and the resultant oxygenation impairment [2]. Therefore, we considered robust immune suppression in the form of high-dose steroids the cornerstone of management tackling both diseases. In addition, plasma exchange therapy, one of the standard treatment modalities for anti-GBM disease, has also been shown to reduce morbidity in critically ill COVID-19 patients [3]. Thus, we promptly started pulsed methylprednisolone and plasmapheresis in our patient. These therapies brought about significant improvement in our patient, with marked clearing of the chest radiograph as well as decrease in oxygen requirement of the patient.

However, the effects of the COVID-19 illness on the pulmonary vasculature in this patient led to a clinical dilemma. The COVID-19-affected lung undergoes fulminant activation of coagulation and consumption of clotting factors [2]. Along with pulmonary capillary endothelitis, these changes lead to microthrombi formation in the pulmonary vasculature, worsening the ventilation-perfusion mismatch in the lungs [2]. As a result, anticoagulation in moderate-to-severe COVID-19 disease is one of the pillars of treatment [4] and is part of the standardised management protocol in our institution. However, DAH is one of the known complications of anti-GBM disease [1], as was seen in our patient. Coupled with the pre-existing thrombocytopenia, this led us to a dilemma over anticoagulation in this patient. Our aim was to minimise the risk of COVID-19-induced pulmonary vascular thrombosis while preventing worsening of DAH caused by anti-GBM disease. As the coagulation profile was normal and there was no clinical evidence of bleeding, we started subcutaneous dalteparin 2500 IU once a day on the second day of ICU stay. Complete hemogram including platelet count and coagulation profile was monitored daily, along with a strict watch on any evidence of bleeding. Platelet count showed no deterioration and coagulation profile remained normal, with no bleeding tendency during the course of hospital stay. However, the patient suffered an episode of haemoptysis, which led to sharp deterioration and eventual death. The underlying frailty of the anti-GBM disease-affected lung, with superimposed inflammatory and vascular compromise caused by COVID-19 disease, may have led to the unfavourable clinical course seen in this patient.

Koc et al. [5] reported the case of an 80-year-old lady with known anti-GBM disease who acquired COVID-19 infection during hospitalisation and succumbed to her illness after 14 days of hospital stay. The authors hypothesised that the poor outcome may have been due to the pre-existing lung pathology induced by anti-GBM disease. Nahhal et al. [6] reported the case of a 63-year-old male who presented with features of DAH and was later diagnosed as anti-GBM disease along with COVID-19 infection. As with our patient, this patient suffered episodes of haemoptysis during ICU stay, deteriorated rapidly, and eventually succumbed. The authors postulated that COVID-19 infection itself can worsen the autoimmune response in susceptible patients, potentially triggering anti-GBM disease. This may be corroborated by the findings of Prendecki et al. [7], who reported an unexpected increased incidence of anti-GBM disease in the United Kingdom, in conjunction with transmission of SARS-CoV-2. The authors attributed this spatial and temporal clustering of cases to the virus triggering an aberrant immune response thus causing anti-GBM disease in those at risk. We can, thus, assume that in patients with pre-existing anti-GBM disease, COVID-19 infection can accelerate the underlying ongoing primary pathology, leading to a more fulminant form of disease. Our patient posed a unique clinical challenge for us in understanding the interaction of these two disease processes and formulating a treatment plan balancing both. Walking the tightrope between undertreating the pulmonary thrombosis and overzealous anticoagulation proved to be difficult in this patient, and may have led to the unfortunate outcome. The complex relationship between the pathophysiology of anti-GBM disease and COVID-19 disease and their combined effect on the lung is still incompletely understood and warrants further research.

## Conclusions

Patients with anti-GBM disease and superimposed COVID-19 infection present a challenge for the treating physician. Both disease processes affect the lung in unique ways. While steroids and immune suppression help to manage both disease processes, the approach towards anticoagulation is less clear-cut. Anticoagulation for management of COVID-19 infection vis-à-vis the risk of DAH from the primary disease process is a fine balance. The decision to give anticoagulants in such patients must be made based on the continuously evolving clinical status. We urge our fellow clinicians to be extremely cautious while giving these patients any form of anticoagulation and keep a low threshold for stoppage of the same.
